# An ethical dilemma: the role of the psychiatrist in physician assisted suicide and/or euthanasia

**DOI:** 10.1192/j.eurpsy.2024.1197

**Published:** 2024-08-27

**Authors:** S. Jesus Magueta, A. L. Costa, G. Simões, A. I. Gomes, C. Madaíl Grego, P. Garrido

**Affiliations:** Departamento de Psiquiatria e Saúde Mental, Centro Hospitalar do Baixo Vouga, Aveiro, Portugal

## Abstract

**Introduction:**

Questions regarding death have generated debates and art since the dawn of civilization. These themes permeate through various areas of study, including religion, philosophy, ethics, medicine and humanities. Various countries have been revising their laws regarding the end of life, especially on the right to aid and choice in the end in the context of medical and phychological suffering. Physician-Assisted Suicide (PAS) and euthanasia are methods by which people, mostly terminal patients, seek to end their lives with the help of medical professionals. PAS and euthanasia have been the target of heated debates in politics and in medicine, with the question of ethics centering most of these.

**Objectives:**

The authors aim to explore PAS and euthanasia in the context of the ethical debate. Based on the pillars of ethics, based on the principal of do no harm and beneficence, the authors explore the role of the Psychiatrist, if any, in these end of life issues.

**Methods:**

The authors performed a brief narrative review of the available literature, with recourse to various databases such as PubMed and Scopus. The search terms utilized in isolation or combination included: *physician assisted suicide, euthanasia, psychiatry, mental illness* and *ethical issues.* Taking into consideration the widespread discussion of these themes in the public forum, news articles were included based on their merit and relevance to the explored topic.

**Results:**

The ethical debate appears to rest between the pillars of first, do no harm, the principles of beneficence and nonmaleficence and aut. Here, the conflict between the first and last appear, where the killing of any patient, whether directly or indirectly is clearly contrary to the principle of primum non nocere. However, the prolonging of suffering in a terminal patient, appears to contradict the principles of nonmaleficence. The Psychiatrist is called to evaluate competence to choose, which is allied to autonomy. Other sources explore the role of the Psychiatrist in permitting a suicide to occur, when the profession is dedicated to the prevention of suicide. From the literature, the psychiatric evaluation is rarely regularly carried out, usually being solicited in cases where mental illness which might compromise the capacity to choose is suspected.

**Conclusions:**

In ethical debates, clear cut answers are rarely every developed, with the nuance and greyscale of difficult topics usually dividing those that ferverantly champion each cause. Psychiatric evaluation is usually invoked when patient autonomy, especially in terms of capacity, is called into question. Questions remain as to whether the presence of the psychiatrist should be a regular one in these procedures or if it should be carried out in a selective manner. There is little consensus in regards to this role, which merits further conversation in the various forums of medical and ethical communication.

**Disclosure of Interest:**

None Declared

